# Higher blood flow rates in anticoagulation-free CRRT improve circuit survival and clinical outcome

**DOI:** 10.1093/ckj/sfaf360

**Published:** 2025-11-21

**Authors:** Caihong Liu, Sifan Fan, Liyao Yang, Wei Wei, Yongxiu Huang, Zhiwen Chen, Qiongxing Bu, Fang Wang, Xue Tang, Yingying Yang, Ping Fu, Ling Zhang, Yuliang Zhao

**Affiliations:** Department of Nephrology, Institute of Kidney Diseases, West China Hospital of Sichuan University, Chengdu, China; Department of Nephrology, West China Hospital of Sichuan University/West China School of Nursing, Sichuan University, Chengdu, China; Department of Nephrology, West China Hospital of Sichuan University/West China School of Nursing, Sichuan University, Chengdu, China; Department of Nephrology, Institute of Kidney Diseases, West China Hospital of Sichuan University, Chengdu, China; Department of Nephrology, Institute of Kidney Diseases, West China Hospital of Sichuan University, Chengdu, China; Department of Nephrology, West China Hospital of Sichuan University/West China School of Nursing, Sichuan University, Chengdu, China; Department of Nephrology, Heping Hospital of Changzhi Medical College, Changzhi, China; Department of Nephrology, West China Hospital of Sichuan University/West China School of Nursing, Sichuan University, Chengdu, China; Department of Nephrology, West China Hospital of Sichuan University/West China School of Nursing, Sichuan University, Chengdu, China; Department of Nephrology, Institute of Kidney Diseases, West China Hospital of Sichuan University, Chengdu, China; Department of Nephrology, Institute of Kidney Diseases, West China Hospital of Sichuan University, Chengdu, China; Department of Nephrology, Institute of Kidney Diseases, West China Hospital of Sichuan University, Chengdu, China; Department of Nephrology, Institute of Kidney Diseases, West China Hospital of Sichuan University, Chengdu, China

**Keywords:** anticoagulation-free, blood flow rate, circuit lifespan, clinical outcome, continuous renal replacement therapy

## Abstract

**Objective:**

Anticoagulation-free continuous renal replacement therapy (CRRT) is an essential modality for managing patients with a high bleeding risk; however, it confronts significant challenges stemming from a shortened circuit lifespan. Blood flow rate (BFR) has been recognized as a pivotal non-pharmacological determinant influencing circuit longevity. This retrospective cohort study aimed to systematically evaluate whether elevating BFR to 250 ml/min could enhance CRRT circuit survival compared with the conventional 200 ml/min in anticoagulation-free settings.

**Methods:**

We conducted a retrospective analysis of patients receiving anticoagulation-free CRRT from seven intensive care units at West China Hospital of Sichuan University between September 2023 and June 2024. The primary outcome was circuit lifespan, defined as the duration from CRRT initiation to termination due to clotting or other causes. Protective and risk factors were assessed using univariate and multivariate Cox proportional hazards regression. Secondary outcomes included the proportion of circuits achieving predefined lifespans and patient clinical outcomes.

**Results:**

Among 128 patients using 241 CRRT circuits (median age: 57 years; 74.27% male), circuits were stratified by BFR: 200 ml/min (*n* = 132) and 250 ml/min (*n* = 109). Survival analysis demonstrated significantly improved 72-hour circuit survival in the 250 ml/min group for both overall circuits (HR: 0.475, 95% CI: 0.329–0.685, *P *< .001), clotted circuits (HR: 0.469, 95% CI: 0.321–0.685, *P *< .001), and each patient’s first circuit (HR: 0.610, 95% CI: 0.373–0.998, *P *= .046), with results remaining statistically significant after confounder adjustment. The 250 ml/min group exhibited longer median circuit lifespans (overall: 33.5 vs. 13 hours; clotted: 31 vs. 15.5 hours; first circuit: 37 vs. 18 hours; all *P *< .05) and higher proportions of circuits achieving predefined lifespans (12 h: 85.32% vs. 65.91%; 24 h: 64.22% vs. 30.30%; 48 h: 33.94% vs. 9.09%; 72 h: 13.76% vs. 3.79%; all *P *< .05). Multivariate analysis identified use of ST150 filter, exposure to non-CRRT anticoagulation agents, and higher activated partial thromboplastin time as protective factors, whereas higher substitute fluid rate, hyperlipidemia, and hematocrit ≥0.30 were associated with reduced circuit survival. Clinically, the 250 ml/min group had lower in-hospital mortality (65.14% vs. 81.06%, *P *= .005). Mediation analysis revealed that higher BFR might prolong circuit lifespan by reducing transmembrane pressure (TMP) increase, which accounted for up to 60.32% of the protective effect.

**Conclusion:**

A BFR of 250 ml/min significantly improved circuit survival in anticoagulation-free CRRT compared to 200 ml/min, likely mediated by TMP reduction. Moreover, patients receiving 250 ml/min BFR exhibited lower in-hospital mortality. Our results support adopting a higher BFR to optimize CRRT efficacy in anticoagulation-free settings. Larger-scale prospective trials are needed to validate the findings.

KEY LEARNING POINTS **What was known:**

- Anticoagulation-free continuous renal replacement therapy (CRRT) is essential for patients at high bleeding risk but is limited by shortened circuit lifespan.
- Blood flow rate (BFR) has been hypothesized to influence circuit patency, yet evidence in anticoagulation-free settings remains sparse and inconsistent.

**This study adds:**

- A BFR of 250 ml/min is associated with significantly longer circuit lifespan (including overall, clotted, and the first circuit per patient) and reduced in-hospital mortality in anticoagulation-free CRRT compared to 200 ml/min.
- Transmembrane pressure (TMP) mediates the beneficial effect of higher BFR on circuit longevity, suggesting a mechanistic link between flow rate and clotting risk.

**Potential impact:**

- This study supports optimizing BFR settings to 250 ml/min in anticoagulation-free CRRT to prolong circuit life and improve patient outcome.
- TMP monitoring may serve as a real-time indicator and interventional target to prevent circuit clotting.

## INTRODUCTION

Continuous renal replacement therapy (CRRT) is a cornerstone therapy for critically ill patients with life-threatening conditions such as acute kidney injury (AKI) and sepsis. The successful performance of CRRT depends on maintaining continuous blood flow through an unobstructed extracorporeal circuit. However, circuit clotting remains a frequent complication, which not only disrupts treatment continuity but also diminishes therapeutic effectiveness, heightens the risk of blood loss, and exacerbates the workload of clinical staff [1–3]. Consequently, considerable research has been devoted to refining anticoagulation strategies to prolong circuit patency [3]. Despite these advances, anticoagulation-free CRRT remains necessary in some high-bleeding-risk patients with the reported utilization rates reaching up to 33%–50% [4, 5]. In such scenarios, circuit survival is notably compromised, with the lifespan typically spanning from 12 to 32 hours, and ∼60% of circuits succumbing to failure within the initial 24 hours [6–10]. These findings underscore the urgent need to identify modifiable factors that can enhance circuit longevity in the absence of anticoagulation.

Emerging evidence suggests that higher blood flow rates (BFR) may prolong extracorporeal circuit lifespan by reducing blood stasis and minimizing the risk of clot formation during CRRT [11]. Notably, a prior analysis demonstrated that a 5.8 ml/min increment in BFR corresponded to an ∼10% improvement in circuit survival [12]. Another study evaluating 1332 treatments in 355 patients found that circuits were significantly more prone to early failure when BFR was maintained below 200 ml/min, with the optimal circuit longevity observed at BFRs between 250 and 300 ml/min [13]. Despite this evidence, considerable variability persists in clinical practice worldwide [11, 13, 14], and more importantly, limited data are available regarding the role of BFR in anticoagulation-free CRRT, where circuit preservation is particularly challenging. In this context, we conducted a retrospective cohort study to evaluate the effect of different BFR levels on circuit lifespan during anticoagulant-free CRRT, thereby seeking to provide evidence-based guidance for optimizing treatment strategies in high-bleeding-risk patients.​

## MATERIALS AND METHODS

### Study design and participants

This retrospective cohort study evaluated adult patients (≥18 years of age) undergoing CRRT from seven intensive care units (ICU) (general ICU, cardiothoracic ICU, medical ICU, surgery ICU, neuro ICU, CCU) at West China Hospital of Sichuan University between 1 September 2023 and 29 June 2024. The exclusion criteria were patients with incomplete clinical documentation for analysis or who were switched to a different anticoagulation strategy or BFR level during CRRT.

Demographic characteristics, CRRT parameters, and relevant laboratory results were extracted from the hospital’s electronic medical record system. The study protocol was approved by the Biomedical Research Ethics Committee of West China Hospital, Sichuan University (approval number 2024-918), and was conducted in accordance with the principles of the Declaration of Helsinki. Given the retrospective nature of the study, patient informed consent was waived.

### CRRT technique

The prescription of CRRT was made jointly by the attending intensivists and CRRT specialists based on a comprehensive clinical assessment. Anticoagulation-free CRRT was preferred for patients with ongoing bleeding or high bleeding risk secondary to coagulation disorders or recent invasive procedures. Although regional citrate anticoagulation (RCA) is generally effective and safe, it was unsuitable for some patients presented with shock, hepatic failure, or hyperlactatemia due to risk of citrate accumulation, or those who had experienced RCA-related adverse events during earlier CRRT sessions, prompting the use of an anticoagulation-free strategy for subsequent treatments. All patients were treated with continuous venous-venous hemodiafiltration (CVVHDF) mode. The CRRT machines used in the study mainly included Prismaflex (Gambro Co. Ltd, Sweden), Plasauto Σ (Sigma Co. Ltd, Japan), and MultiFiltrate (Fresenius Co. Ltd, Germany). The filters primarily used included ST150, oXiris, and 180 W. Depending on the clinical scenario and equipment availability, both post-dilution and combined pre-/post-dilution methods were employed. Prescribed CRRT treatment doses ranged from 25 to 35 ml/kg/h. The dialysate was set at 1000 ml/h. Vascular access was established under ultrasound guidance, typically via uncuffed dual-lumen catheters inserted into the internal jugular or femoral veins. In accordance with manufacturer recommendations, filters were routinely replaced every 72 hours to ensure sustained therapeutic efficacy. During anticoagulation-free CRRT, BFR were set at either 200 ml/min or 250 ml/min, which was jointly determined by CRRT specialists and ICU physicians based on a comprehensive clinical assessment. Specifically, a BFR of 250 ml/min was preferred for patients with well-functioning vascular access, stable hemodynamics, and acceptable arterial and venous pressures without alarms. Conversely, a BFR of 200 ml/min was selected when any of these conditions were suboptimal.

### Outcomes and measurement

The primary outcome was circuit lifespan, assessed across all circuits, clotted circuits, and the first circuit used per patient. The circuit lifespan was measured in hours and calculated from single CRRT initiation to the termination due to circuit clotting or ceased due to other reasons, such as scheduled treatment completion, routine filter replacement at 72 hours, and patient death. The potential protective and risk factors influencing the CRRT circuit survival were identified. The secondary outcomes included the followings: (i) the proportion of circuits achieving predefined lifespan milestones (12 hours, 24 hours, 48 hours, and 72 hours); (ii) clinical outcomes, including the incidence of invasive mechanical ventilation, length of hospitalization, in-hospital mortality and dialysis dependency at discharge; (iii) dynamic changes in circuit pressures at different timepoints after CRRT initiation, including arterial pressure (AP), venous pressure (VP), and transmembrane pressure (TMP).

### Statistical analysis

Continuous variables were expressed as means ± standard deviations (SD) or as medians with interquartile ranges (IQR), contingent on their distribution. Categorical variables were presented as frequencies and percentages. Inter-group differences for categorical variables were analyzed using chi-square (*χ*²) or Fisher’s exact tests, as appropriate. For continuous data, comparisons were conducted using Student’s *t*-tests for normally distributed variables or Wilcoxon rank-sum tests for variables exhibiting non-normal distributions. Time-to-event data for circuit termination were analyzed using Kaplan–Meier (K–M) survival curves and compared between groups using the log-rank test. Hazard ratios (HRs) with 95% confidence intervals (CIs) were estimated. Univariate Cox regression analyses were conducted to evaluate the effect of individual patient characteristics [e.g. age, sex, body mass index (BMI), laboratory parameters, and CRRT-related variables] on circuit lifespan. Variables with statistical significance in univariate analyses were included into a multivariate Cox regression model to identify independent protective and risk factors. Three analytical models were constructed: Model 1 was unadjusted; Model 2 was adjusted for age, sex, and BMI; Model 3 was further adjusted for baseline covariates that significantly differed between groups (*P* < .05). To avoid multicollinearity and overfitting, correlated variables were excluded from the multivariable models. Subgroup analyses were additionally performed to identify subpopulations deriving greater benefit from specific BFR settings. Logistic regression models were employed to evaluate the association between circuit pressures and circuit clotting events. The relationships among continuous variables (circuit pressure, BFR, and clotted circuit lifespan) were assessed using Pearson or Spearman correlation tests, depending on data distribution. To investigate potential causal pathways, mediation analysis was conducted with BFR as the independent variable, circuit pressure as the mediator, and circuit lifespan as the outcome. The mediation R package (version 4.3) was used to decompose total effects into direct and indirect effects, with bias-corrected 95% CIs estimated via 1000 bootstrap iterations. For variables with missing data less than 10%, median imputation was used, while those larger than 10% were excluded from the analysis. All the statistical analyses were performed with R software (version 4.3; R Foundation for Statistical Computing, Vienna, Austria). A two-tailed *P *< .05 was considered to indicate statistical significance.

## RESULTS

### Baseline characteristics

During the study period, 128 patients who underwent anticoagulation-free CRRT with a total of 241 extracorporeal circuits were included in the analysis. Of these, 132 circuits were in the 200 ml/min group and 109 in the 250 ml/min group. All CRRT circuits were operated in adherence to anticoagulation-free protocols, with BFR maintained at predetermined levels for the duration of therapy. Baseline demographic and clinical characteristics are summarized in Table 1. Patient demographics were generally balanced between the two groups. The median age was 57 years, with a median BMI of 23.2 kg/m², and 74.27% of patients were male. The predominant indication for CRRT was AKI (39%), either alone or in combination with other conditions such as sepsis, followed by chronic renal failure (33.2%). The most commonly used filter and machine type in our center were ST150 (68.05%) and Prismaflex (90.04%), respectively. Vascular access was primarily via the femoral vein (87.97%), and pre-/post-dilution was used in 79.67% of the treatment sessions. In comparison to the 250 ml/min group, circuits in the 200 ml/min group experienced higher filtration fraction (FF) (0.15 vs. 0.09), therapeutic dose (33.27 vs. 27.02 ml/kg/h), substitution fluid rate (1000 vs. 700 ml/h), and sodium bicarbonate infusion rate (140 vs. 100 ml/h) (all *P *< .05). A higher proportion of patients in the 250 ml/min group received anticoagulation agents for non-CRRT purpose during hospitalization (41.28% vs. 15.15%, *P *< .01). With regard to the baseline laboratory tests, patients in the 250 ml/min group had more favorable coagulation profiles, including elevated platelet (131 vs. 92.5*10^9^/l), fibrinogen (FIB) (3.09 vs. 2.28 g/l), and shorter thrombin time (TT) (17.3 vs 18.5 s). Additionally, the triglyceride (TG) (2.57 vs. 1.51 mmol/l), blood urea nitrogen (BUN) (18.6 vs. 12.7 mmol/l), and uric acid (UA) (468 vs. 378μmol/l) were higher in the 250 ml/min group (all *P *< .05). As for disease severity, the requirements for vasopressor and mechanical ventilation were similar (28.79% vs. 23.85%, *P *= .388, and 41.67% vs. 33.94%, *P *= .219, respectively) between the two BFR groups. But the baseline lactate level was slightly higher in the 200 ml/min BFR group (3.7 vs. 2.4 mmol/l, *P *= .004). No significant differences were observed for other baseline data.

**Table 1:** Baseline characteristics of patients and circuits.

Variables	Total (*n* = 241)	200 ml/min (*n* = 132)	250 ml/min (*n* = 109)	Statistic	*P*
Age, years	57.00 (48.00, 71.00)	62.00 (47.50, 71.00)	52.00 (48.00, 72.00)	*Z* = −1.53	.126
Gender, *n* (%)				*χ*² = 3.11	.078
Female	62 (25.73)	28 (21.21)	34 (31.19)		
Male	179 (74.27)	104 (78.79)	75 (68.81)		
BMI, kg/m^2^	23.20 (20.80, 25.26)	23.05 (20.80, 25.10)	24.50 (21.03, 25.64)	*Z* = −1.39	.163
CRRT parameters					
CRRT indication, *n* (%)				*χ*² = 11.53	.003
AKI	94 (39)	63 (47.73)	31 (28.44)		
Chronic renal failure	80 (33.20)	42 (31.82)	38 (34.86)		
Non-renal	67 (27.80)	27 (20.45)	40 (36.70)		
Filter type, *n* (%)					.002
180W	15 (6.22)	2 (1.52)	13 (11.93)		
AV1000s	6 (2.49)	5 (3.79)	1 (0.92)		
AV600	4 (1.66)	4 (3.03)	0 (0.00)		
Oxiris	52 (21.58)	29 (21.97)	23 (21.10)		
ST150	164 (68.05)	92 (69.70)	72 (66.06)		
Machine type, *n* (%)					.002
MultiFiltrate	7 (2.90)	6 (4.55)	1 (0.92)		
Prismaflex	217 (90.04)	121 (91.67)	96 (88.07)		
Aquarius	4 (1.65)	3 (2.27)	1 (0.92)		
Plasauto Σ	13 (5.39)	2 (1.52)	11 (10.09)		
Access, *n*(%)					.122
Femoral vein	212 (87.97)	120 (90.91)	92 (84.40)		
Jugular vein	29 (12.03)	12 (9.09)	17 (15.60)		
Dilution, *n* (%)				*χ*² = 22.77	<.001
Post-dilution	49 (20.33)	12 (9.09)	37 (33.94)		
Pre- and post-dilution	192 (79.67)	120 (90.91)	72 (66.06)		
Ultrafiltration					
Ultrafiltration, ml	1600.00 (600.00, 3650.00)	1200.00 (197.50, 2350.00)	2975.00 (860.00, 5375.00)	*Z* = *−*4.86	<.001
Net ultrafiltration rate, ml/kg/h	1.24 (0.64, 2.05)	1.25 (0.50, 2.46)	1.21 (0.79, 1.80)	*Z* = −0.70	.485
Filtration fraction	0.12 (0.09, 0.16)	0.15 (0.13, 0.17)	0.09 (0.08, 0.10)	*Z* = *−*12.46	<.001
Therapeutic dose, ml/kg/h	29.72 (26.46, 36.00)	33.27 (29.30, 38.04)	27.02 (24.42, 30.51)	*Z* = −6.88	<.001
Substitution rate, ml/h	1000.00 (700.00, 1000.00)	1000.00 (1000.00, 1000.00)	700.00 (700.00, 750.00)	*Z* = *−*11.52	<.001
Sodium bicarbonate rate, ml/h	125.00 (100.00, 160.00)	140.00 (125.00, 160.00)	100.00 (90.00, 120.00)	*Z* = *−*8.51	<.001
First circuit, *n* (%)	128 (53.11)	72 (54.55)	56 (51.38)	*χ*²=0.24	.624
Concomitant therapy					
Anti-platelet agents	11 (4.56)	4 (3.03)	7 (6.42)	*χ*² = 0.89	.344
Anticoagulation agents	65 (26.97)	20 (15.15)	45 (41.28)	*χ*²=20.70	<.001
Laboratory tests					
HB, g/l	90.00 (78.00, 117.00)	83.00 (74.75, 124.00)	92.00 (83.00, 109.00)	*Z* = *−*1.05	.294
Hct, l/l	0.28 (0.23, 0.36)	0.28 (0.23, 0.39)	0.28 (0.24, 0.33)	*Z* = *−*0.26	.794
WBC, *10^9^/l	11.04 (6.99, 16.40)	9.50 (6.73, 16.38)	12.45 (7.33, 18.07)	*Z* = *−*1.70	.089
Platelet, *10^9^/l	116.00 (60.00, 178.00)	92.50 (37.00, 173.00)	131.00 (76.00, 185.00)	*Z* = *−*3.19	.001
APTT, s	32.30 (28.30, 40.40)	31.75 (27.50, 40.65)	33.00 (29.60, 39.80)	*Z* = *−*0.98	.329
PT, s	15.30 (12.80, 18.90)	15.60 (12.62, 18.35)	14.90 (13.30, 23.60)	*Z* = *−*1.33	.184
TT, s	17.70 (16.30, 21.70)	18.50 (16.40, 22.60)	17.30 (16.10, 21.10)	*Z* = *−*2.36	.018
INR	1.33 (1.14, 1.68)	1.39 (1.13, 1.64)	1.28 (1.14, 1.92)	*Z* = *−*0.46	.646
D-dimer, mg/l FEU	7.03 (2.76, 14.31)	7.03 (2.39, 11.29)	7.15 (3.12, 20.08)	*Z* = *−*0.70	.487
FIB, g/l	3.00 (1.89, 4.77)	2.28 (1.45, 4.65)	3.09 (2.29, 5.22)	*Z* = *−*2.79	.005
TP, g/l	59.40 (53.40, 64.80)	60.70 (55.00, 66.50)	57.60 (52.20, 64.00)	*Z* = *−*1.84	.065
ALB, g/l	32.80 (29.40, 37.40)	32.30 (28.00, 35.50)	34.20 (30.60, 38.80)	*Z* = *−*2.55	.011
ALT, U/l	26.00 (13.00, 92.00)	24.00 (13.00, 81.00)	31.00 (11.00, 96.00)	*Z* = *−*0.74	.461
AST, U/l	52.00 (22.00, 117.00)	54.00 (23.00, 93.25)	51.00 (20.00, 139.00)	*Z* = *−*0.37	.714
ALP, U/l	90.00 (70.00, 156.00)	87.50 (63.25, 131.25)	98.00 (74.00, 170.00)	*Z* = *−*1.78	.074
TG, mmol/l	1.73 (1.00, 3.22)	1.51 (0.96, 2.56)	2.57 (1.21, 3.22)	*Z* = *−*3.20	.001
Chol, mmol/l	2.08 (1.27, 3.47)	2.08 (1.40, 3.45)	2.27 (1.18, 3.53)	*Z* = *−*0.01	.990
Scr, μmol/l	251.00 (107.00, 408.00)	191.50 (82.75, 408.00)	294.00 (110.00, 391.00)	*Z* = *−*1.93	.054
BUN, mmol/l	14.10 (7.70, 22.90)	12.70 (7.10, 21.50)	18.60 (10.30, 26.90)	*Z* = *−*3.23	.001
eGFR, ml/min/1.74 m^2^	21.86 (9.88, 66.50)	27.48 (9.81, 85.09)	15.85 (10.14, 61.67)	*Z* = *−*1.63	.104
CystatinC, mg/l	2.81 (1.40, 4.96)	2.46 (1.50, 5.13)	3.32 (1.33, 3.86)	*Z* = *−*0.10	.919
UA, umol/l	410.00 (272.00, 634.00)	378.00 (247.50, 593.50)	468.00 (328.00, 658.00)	*Z* = *−*2.45	.014
Disease severity					
Lactate, mmol/l	3.10 (1.84, 6.16)	3.70 (2.17, 6.70)	2.40 (1.65, 4.72)	*Z* = −3.01	.004
Vasopressor requirement, *n* (%)	64 (26.56)	38 (28.79)	26 (23.85)	*χ*² = 0.75	.388
Mechanical ventilation, *n* (%)	92 (38.17)	55 (41.67)	37 (33.94)	*χ*² = 1.51	.219

Abbreviations: HB, hemoglobin; WBC, white blood cell; PT, prothrombin time; INR, international normalized ratio; FIB, fibrinogen; TP, total protein; ALB, albumin; ALT, alanine aminotransferase; AST, aspartate aminotransferase; ALP, alkaline phosphatase; TG, triglyceride; Chol, cholesterol; Scr, serum creatine.

## CIRCUIT LIFESPAN

### Comparison of overall circuit lifespan

**Figure 1:**
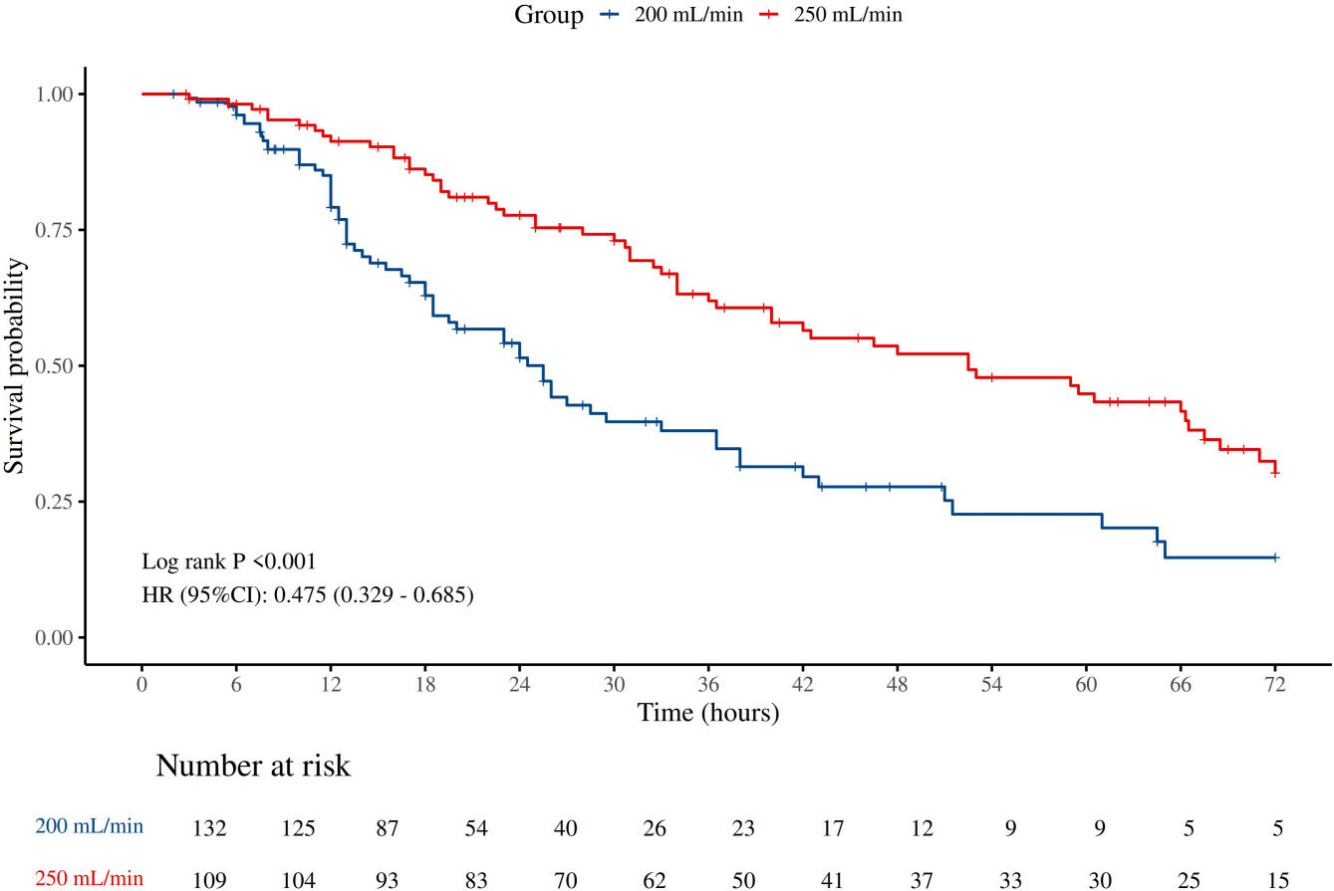
The K–M curve for the overall circuit’s 72-hour survival.

In anticoagulation-free CRRT, circuits operated at a BFR of 250 ml/min demonstrated a significantly longer overall median lifespan compared to those operated at 200 ml/min (33.5 vs. 13 hours, *P *< .001) (Table 2). The K–M curve further confirmed that higher BFR was associated with superior 72-hour circuit survival (HR = 0.475, 95%CI 0.329–0.685, *P *< .001) (Fig. 1). In Cox regression survival analysis, consistent results were demonstrated after adjusting for baseline demographics and confounders in Model 2 (HR = 0.52, 95%CI 0.35–0.76, *P *< .001) and Model 3 (HR = 0.49, 95%CI 0.29–0.84, *P *= .009) (Table 3). Moreover, circuits in the 250 ml/min group were also significantly more likely to achieve predefined lifespan milestones. Specifically, 13.76% of circuits in the 250 ml/min group reached the lifespan of 72 hours, compared to only 3.79% in the 200 ml/min group (*P *= .005). Similarly, circuit patency beyond 12 hours (85.32% vs. 65.91%), 24 hours (64.22% vs. 30.30%), and 48 hours (33.94% vs. 9.09%) was consistently better in the 250 ml/min group than in the 200 ml/min (all *P *< .001) (Table 2). Subgroup analyses identified potentially significant interaction effects by sex and age. Males and patients under 65 years of age seemed to experience greater benefit from higher BFR levels in terms of circuit survival (*P* for interaction: 0.019, 0.042, respectively) (Table 4).

**Table 2:** Circuit lifespan and clinical outcomes between the 200 ml/min and 250 ml/min groups.

Variables	Total (*n* = 241)	200 ml/min (*n* = 132)	250 ml/min (*n* = 109)	Statistic	*P*
Overall circuit lifespan, hours	20.00 (11.50, 40.00)	13.00 (8.00, 25.50)	33.50 (18.50, 62.00)	*Z* = −5.95	<.001
Ideal circuit lifespan percent					
12 hours, *n* (%)	180 (74.69)	87 (65.91)	93 (85.32)	*χ*² = 11.90	<.001
24 hours, *n* (%)	110 (45.64)	40 (30.30)	70 (64.22)	*χ*² = 27.68	<.001
48 hours, *n* (%)	49 (20.33)	12 (9.09)	37 (33.94)	*χ*² = 22.77	<.001
72 hours, *n* (%)	20 (8.30)	5 (3.79)	15 (13.76)	*χ*² = 7.80	.005
Clinical outcomes					
Invasive mechanical ventilation, *n* (%)	95 (39.42)	53 (40.15)	42 (38.53)	*χ*² = 0.07	.798
In-hospital death, *n* (%)	178 (73.86)	107 (81.06)	71 (65.14)	*χ*² = 7.84	.005
First circuit, *n* (%)	128 (53.11)	72 (54.55)	56 (51.38)	*χ*² = 0.24	0.624
First circuit lifespan, h	23.75 (12.38, 47.62)	18.00 (12.00, 32.78)	37.00 (19.00, 64.25)	*Z* = −3.70	<0.001
Clotted, *n* (%)	122 (50.62)	67 (50.76)	55 (50.46)	*χ*² = 0.00	.410
Filter, *n* (%)	104 (85.25)	56 (83.58)	48 (87.27)		
Tube, *n* (%)	3 (2.46)	3 (4.48)	0 (0.00)		
Venous chamber, *n* (%)	15 (12.30)	8 (11.94)	7 (12.73)		
Clotted circuit lifespan, h	19.75 (12.00, 36.38)	15.50 (11.25, 25.75)	31.00 (17.50, 47.25)	*Z* = −3.81	<.001
Survived hospitalization, *n* (%)	63 (26.14)	25 (18.94)	38 (34.86)	*χ*² = 7.84	.005
Dialysis dependency, *n* (%)	47 (74.60)	22 (88.00)	25 (65.79)	*χ*² = 3.93	.048
Length of hospitalization, days	23.00 (23.00, 34.50)	23.00 (23.00, 23.00)	25.50 (23.00, 42.00)	*Z* = −2.79	.005

**Table 3:** Multivariate Cox proportional hazard model for overall, clotted, and the first circuit lifespan based on BFR levels.

	Model 1		Model 2		Model 3	
Variables	HR (95%CI)	*P*	HR (95%CI)	*P*	HR (95%CI)	*P*
Overall circuit lifespan						
200 ml/min	1.00 (Reference)		1.00 (Reference)		1.00 (Reference)	
250 ml/min	0.47 (0.33–0.68)	<.001	0.52 (0.35–0.76)	<.001	0.49 (0.29–0.84)	.009
Clotted circuit lifespan						
200 ml/min	1.00 (Reference)		1.00 (Reference)		1.00 (Reference)	
250 ml/min	0.47 (0.32–0.69)	<.001	0.47 (0.31–0.70)	<.001	0.44 (0.25–0.76)	.003
First circuit lifespan						
200 ml/min	1.00 (Reference)		1.00 (Reference)		1.00 (Reference)	
250 ml/min	0.61 (0.37–0.99)	.049	0.68 (0.40–1.14)	.142	0.48 (0.23–0.99)	.046

Model 1: unadjusted model

Model 2: adjusted by age, gender, and BMI

Model 3: adjusted by age, gender, BMI, filter type, CRRT indication, use of anticoagulation agents, FF, TT, TG, BUN, UA, and baseline lactate

**Table 4:** Univariate and multivariate analyses of potential protective and risk factors for circuit lifespan.

	Univariate model		Multivariate model	
Variables	HR (95%CI)	*P*	HR (95%CI)	*P*
Age	1.01 (0.99–1.02)	.318		
Gender				
Female	1.00 (Reference)			
Male	0.83 (0.56–1.24)	.363		
CRRT parameters				
BFR				
200 ml/min	1.00 (Reference)		1.00 (Reference)	
250 ml/min	0.47 (0.33–0.68)	<.001	0.56 (0.34–0.91)	.019
CRRT indication				
AKI	1.00 (Reference)		1.00 (Reference)	
Chronic renal failure	1.43 (0.94–2.17)	.092		
Non-renal	0.82 (0.52–1.28)	.372		
Filter type				
180W	1.00 (Reference)		1.00 (Reference)	
AV1000s	0.64 (0.18–2.27)	.488	0.78 (0.21–2.97)	.717
AV600	4.16 (0.87–19.83)	.074	2.45 (0.48–12.59)	.283
Oxiris	0.38 (0.18–0.77)	.008	0.53 (0.24–1.17)	.116
ST150	0.43 (0.23–0.80)	.007	0.42 (0.22–0.81)	.009
Machine type				
MultiFiltrate	1.00 (Reference)			
Prismaflex	0.51 (0.19–1.39)	.188		
Aquarius	2.06 (0.23–18.70)	.521		
PlasautoΣ	1.12 (0.35–3.59)	.847		
Access				
Femoral vein		1.00 (Reference)		
Jugular vein	0.95 (0.55–1.63)	.856		
Dilution				
Post-dilution	1.00 (Reference)			
Pre-and Post-dilution	0.72 (0.48–1.08)	.115		
Ultrafiltration				
Net ultrafiltration rate	1.15 (0.92–1.43)	.218		
Therapeutic dose	1.06 (1.03–1.09)	<.001	0.96 (0.77–1.19)	.705
Substitute rate	1.01 (1.01–1.01)	<.001	1.01 (1.01–1.01)	.002
Sodium bicarbonate rate	1.01 (1.01–1.01)	.025	1.00 (0.99–1.00)	.424
First circuit	0.82 (0.57–1.17)	.263		
Concomitant therapy				
Anti-platelet agents	0.84 (0.34–2.05)	.695		
Anticoagulation agents	0.41 (0.26–0.63)	<.001	0.62 (0.39–0.99)	.049
Comorbidities				
Hypoalbumin	1.28 (0.86–1.89)	.219		
Hypotension	1.18 (0.81–1.72)	.376		
Obesity	0.97 (0.92–1.02)	.219		
Live Dysfunction	0.88 (0.61–1.29)	.526		
Hyperlipidemia	2.68 (1.24–5.81)	.012	4.02 (1.76–9.20)	<.001
Hyperuricemia	1.01 (0.71–1.44)	.956		
Acidosis	0.93 (0.65–1.33)	.700		
Hyperlactataemia	0.71 (0.41–1.21)	.208		
Laboratory tests				
Baseline				
HB, g/l	1.00 (1.00–1.01)	.325		
Hct				
<0.3		1.00 (Reference)		
≥0.3	1.28 (0.90–1.84)	.171		
WBC	0.99 (0.98–1.01)	.377		
Platelet	1.00 (1.00–1.00)	.679		
APTT	1.00 (0.99–1.01)	.998		
INR	1.12 (0.94–1.33)	.197		
D-dimer	1.00 (0.99–1.02)	.714		
Scr	1.01 (1.01–1.01)	.009		
Before CRRT				
Platelet	1.00 (1.00–1.00)	.409		
APTT	0.99 (0.98–0.99)	.038	0.99 (0.98–0.99)	.035
INR	0.93 (0.79–1.09)	.353		
D-dimer	1.00 (0.99–1.02)	.634		
Hct				
<0.3		1.00 (Reference)		
≥0.3	1.84 (1.18–2.87)	.007	2.29 (1.43–3.65)	<.001

Abbreviations: HB, hemoglobin; WBC, white blood cell; INR, international normalized ratio; Scr, serum creatine.

### Comparison of clotted circuit lifespan

**Figure 2:**
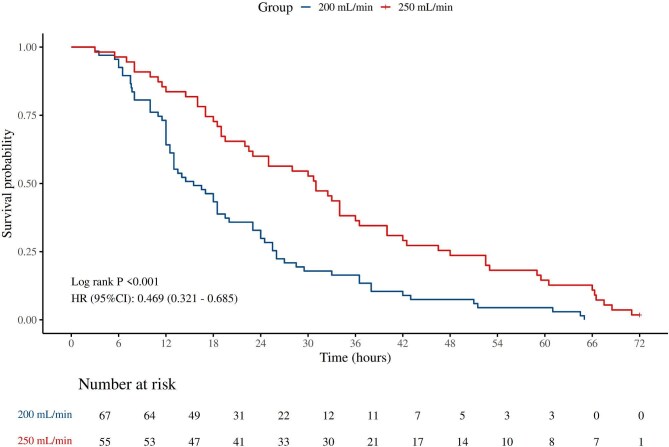
The K–M curve for the clotted circuit’s 72-hour survival.

Of the 241 circuits, 122 (50.62%) were terminated due to clotting, the filter was identified as the predominant site of clot formation, accounting for 85.25% of clotting events. The K–M curve also showed that higher BFR conferred a better 72-hour circuit survival (HR: 0.469, 95% CI: 0.321–0.685, *P *< .001) (Fig. 2). The result was also statistically significant by adjusting for potential interferences in Model 2 (HR: 0.47, 95% CI: 0.31–0.70, *P *< .001) and Model 3 (HR: 0.44, 95% CI: 0.25–0.76, *P *= .003) (Table 3). The median lifespan of clotted circuits was significantly longer in the higher BFR group (31 vs. 15.5 hours, *P *< .001), indicating a delayed onset of clotting with higher BFR at 250 ml/min.

### Comparison of the first circuit lifespan per patient

**Figure 3:**
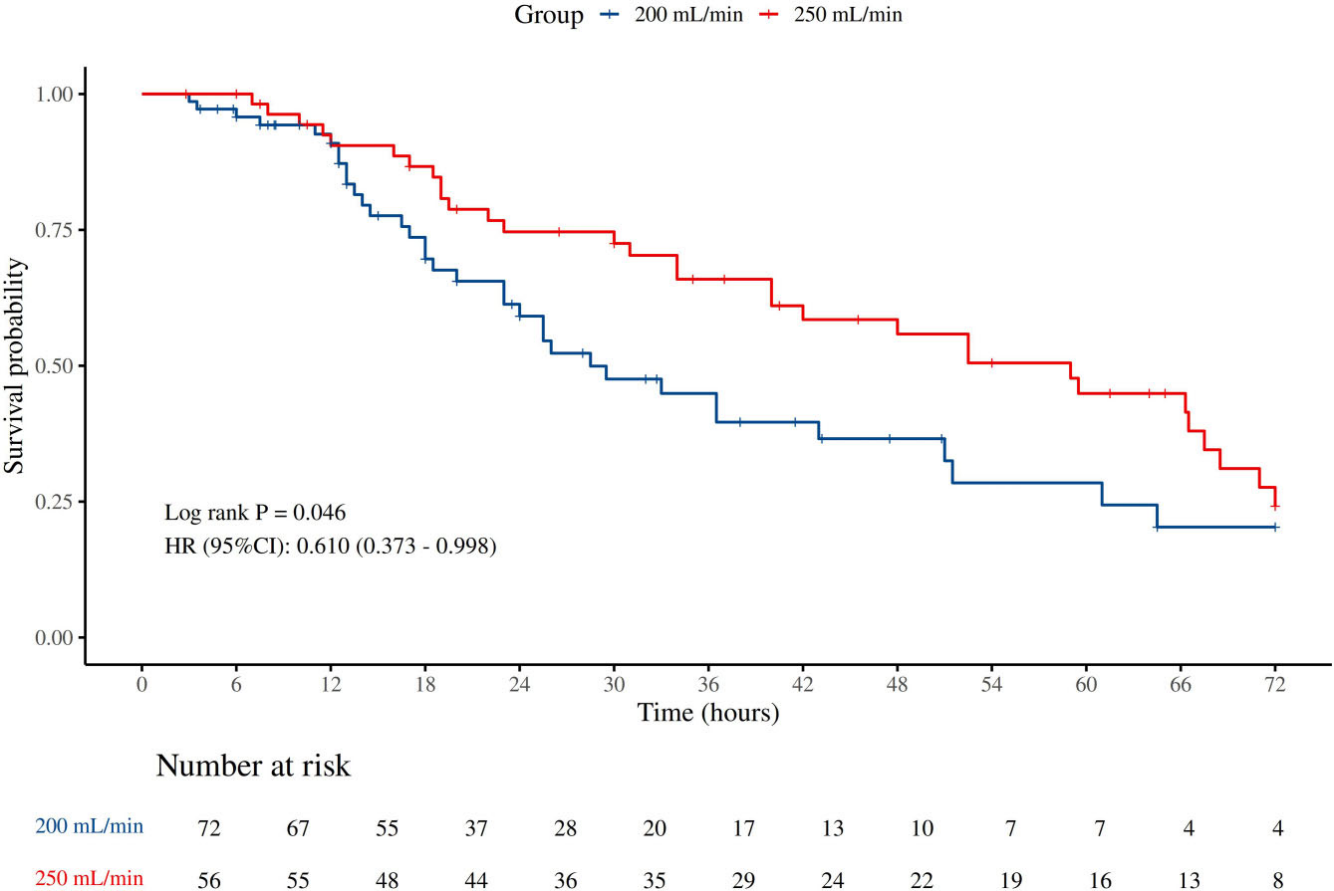
The K–M curve for the first circuit’s 72-hour survival.

For each patient’s first circuit (*n* = 128), the 250 ml/min group also demonstrated superior performance, with a median lifespan of 37.0 hours compared to 18.0 hours in the 200 ml/min group (*P *< .001), and a similar result was displayed by K–M analysis (HR = 0.610, 95%CI 0.373–0.998, *P *= .046) (Fig. 3). In multivariate Cox regression, the association remained significant in the fully adjusted Model 3 (HR = 0.48, 95%CI 0.23–0.99, *P *= .046), although it did not reach statistical significance in Model 2 (HR = 0.68, 95%CI 0.40–1.14, *P *= .142) (Table 3).

### Other independent influencing factors for circuit lifespan

Multivariate Cox regression identified several independent predictors of circuit lifespan other than BFR (Table 5). Higher substitution fluid rate, presence of hyperlipidemia, and hematocrit (Hct) ≥0.30 were associated with poorer circuit patency. Use of ST150 filter, concomitant use of non-CRRT anticoagulation agents, and higher activated partial thromboplastin time (APTT) were associated with improved survival of circuits.

**Table 5:** Subgroup analysis of the impact of BFR on circuit lifespan.

Variables	*n* (%)	200 ml/min	250 ml/min	HR (95%CI)	*P*	*P* for interaction
All patients	241 (100.00)	67/132	55/109	0.47 (0.33–0.68)	<.001	
Gender						.019
Female	62 (25.73)	13/28	22/34	0.97 (0.49–1.94)	.938	
Male	179 (74.27)	54/104	33/75	0.36 (0.23–0.56)	<.001	
Age, years						.042
>65	93 (38.59)	28/62	18/31	0.89 (0.49–1.61)	.694	
≤65	148 (61.41)	39/70	37/78	0.35 (0.22–0.56)	<.001	
Filter type						.241
180W	15 (6.22)	2/2	10/13	0.66 (0.14–3.21)	.610	
AV1000s	6 (2.49)	3/5	0/1	0.00 (0.00–Inf)	.999	
AV600	4 (1.66)	2/4	0/0			
Oxiris	52 (21.58)	11/29	9/23	0.57 (0.23–1.40)	.218	
ST150	164 (68.05)	49/92	36/72	0.42 (0.27–0.65)	<.001	
Machine type						.105
MultiFiltrate	7 (2.90)	4/6	0/1	0.00 (0.00–Inf)	.999	
Prismaflex	217 (90.04)	60/121	46/96	0.45 (0.30–0.67)	<.001	
Aquarius	4 (1.65)	1/3	1/1			
PlasautoΣ	13 (5.39)	2/2	8/11	0.61 (0.12–3.15)	.553	
Access						.175
Femoral vein	212 (87.97)	61/120	46/92	0.50 (0.34–0.74)	<.001	
Jugular vein	29 (12.03)	6/12	9/17	0.19 (0.05–0.68)	.010	
CRRT indication						.499
AKI	94 (39.00)	36/63	14/31	0.33 (0.17–0.63)	<.001	
Chronic renal failure	80 (33.20)	17/42	24/38	0.55 (0.28–1.07)	.080	
Non-renal	67 (27.80)	14/27	17/40	0.50 (0.24–1.02)	.050	
Anti-platelet agents						.668
No	230 (95.44)	65/128	52/102	0.48 (0.33–0.70)	<.001	
Yes	11 (4.56)	2/4	3/7	0.20 (0.02–2.33)	.200	
Anticoagulation agents						.339
No	176 (73.03)	57/112	38/64	0.65 (0.43–0.99)	.043	
Yes	65 (26.97)	10/20	17/45	0.39 (0.18–0.85)	.019	

### Clinical outcomes

As charted in Table 2, patients in the 250 ml/min BFR group exhibited a significantly lower in-hospital mortality rate compared to those in the 200 ml/min group (65.14% vs. 81.06%, *P *= .005). The proportion of patients requiring invasive mechanical ventilation did not differ significantly between groups (38.53% vs. 40.15%, *P* = .798). Among survivors, a reduced proportion of patients in the 250 ml/min BFR group remained dialysis-dependent at discharge (65.79% vs. 88%, *P *= .048). However, patients in the 250 ml/min group had a slightly longer hospital stay (25.5 vs. 23 days, *P *= .005). After adjustment by baseline disease severity encompassing lactate, vasopressor requirement, and mechanical ventilation, only the in-hospital mortality remained statistically significant (OR = 0.32, 95%CI 0.17–0.60, *P *< .001), while there was no difference regarding dialysis dependency (OR = 3.43, 95%CI 0.79–14.87, *P *= .100) and length of hospitalization (*β* = 6.36, 95%CI −1.51–14.24, *P *= .119) among those who survived hospital between the high-BFR group and the low-BFR group (Supplementary Table 1).

### Impact of BFR-associated circuit pressure dynamics on circuit clotting

**Figure 4:**
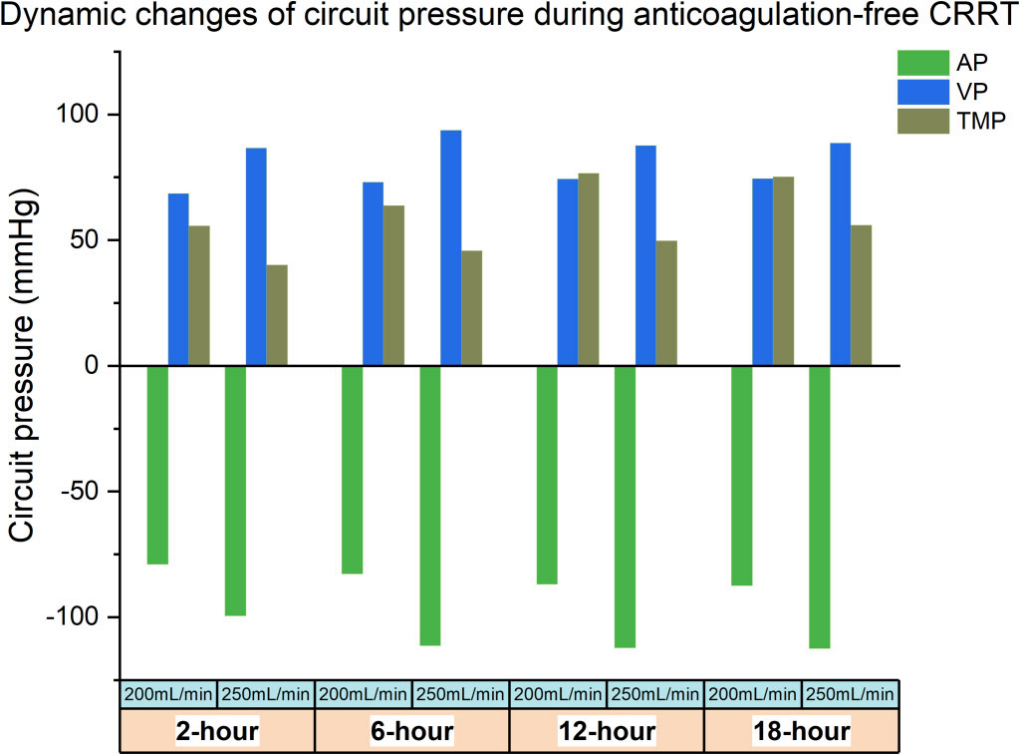
Dynamic changes of AP, VP, and TMP during CRRT.

**Figure 5:**
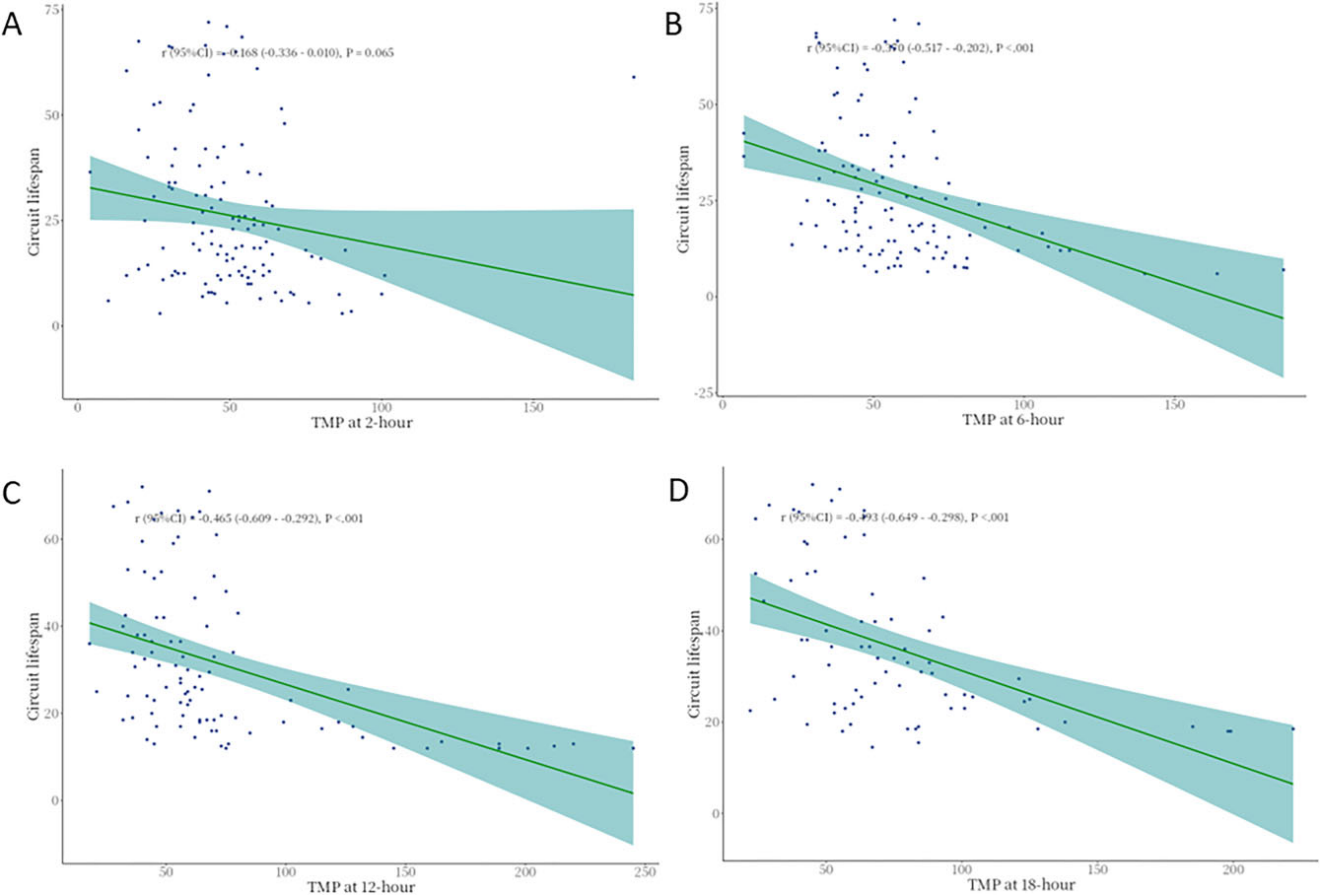
Scatter plot demonstrating the association between TMP and clotted circuit lifespan. A: The association between TMP at 2-hour and clotted circuit life; B: The association between TMP at 6-hour and clotted circuit life; C: The association between TMP at 12-hour and clotted circuit life; D: The association between TMP at 18-hour and clotted circuit life.

We further investigated the dynamic changes in circuit pressures and their potential relationship with clotting risk. At higher BFR (250 ml/min), the absolute value of both AP and VP were significantly elevated compared to lower BFR across timepoints (all *P *< .001), with AP reaching −136 vs. −98 mmHg and VP 116 vs. 86.5 mmHg at peak values (Fig. 4 and Supplementary Table 1). While these pressure increases might raise the likelihood of circuit alarms, TMP exhibited an inverse pattern, remaining consistently lower in the high-BFR group at all observed timepoints (all *P *< .001). This lower TMP was clinically significant as it correlated with reduced clotting risk, evidenced by both logistic regression (e.g. 18-hour OR = 1.03, 95%CI 1.01–1.05, *P *< .001) and progressively stronger negative correlations between TMP and circuit survival over time (e.g. 18-hour *r* = −0.493, 95%CI −0.649–0.298, *P *< .001) (Fig. 5, Supplementary Table 2). Further mediation analysis revealed that the protective effect of high BFR at 250 ml/min on circuit lifespan was partially mediated through TMP reduction, with the mediation effect accounting for 60.32% of the total benefit at 12 hours (*β* = 15.03, *P *< .001), although this proportion decreased to 37.62% by 18 hours. Overall, higher BFR was associated with a more negative AP, a higher VP, and a modest reduction in TMP, and the decreased TMP may contribute to prolonged circuit survival (Fig. 5).

**Figure 6:**
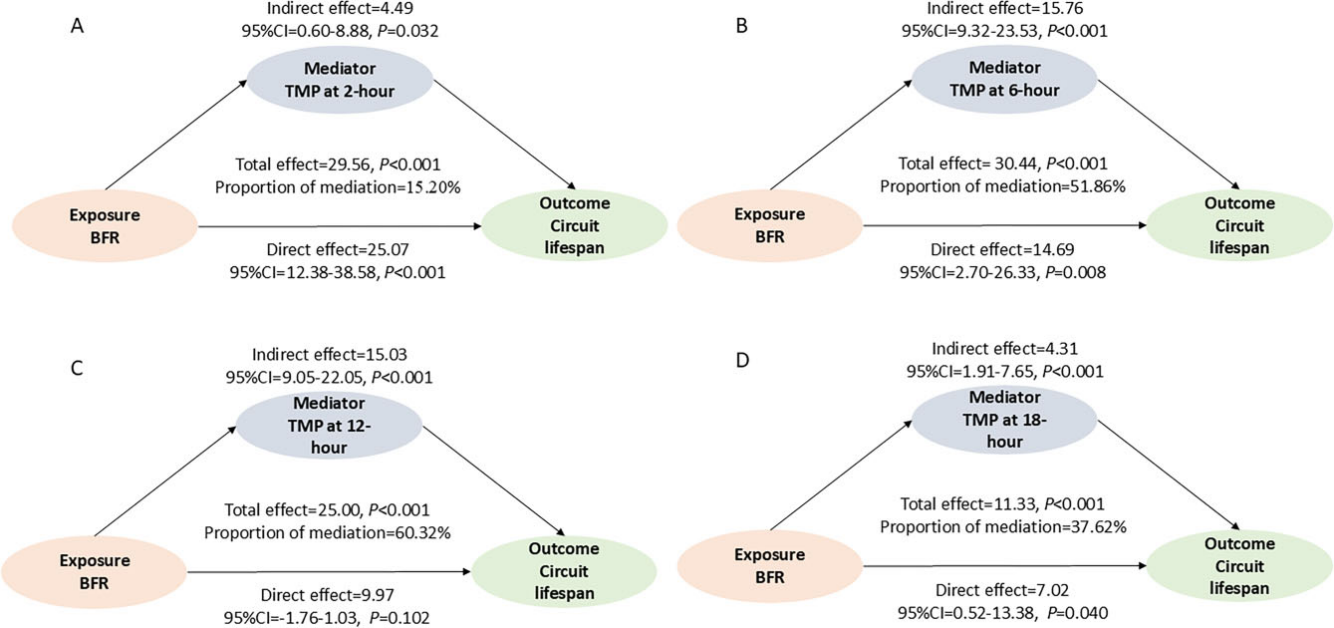
Mediation analysis for the guiding role of TMP on shortened circuit lifespan. Panel A: Mediation analysis between TMP at 2-hour on shorted circuit lifespan; Panel B: Mediation analysis between TMP at 6-hour on shorted circuit lifespan; Panel C: Mediation analysis between TMP at 12-hour on shorted circuit lifespan; Panel D: Mediation analysis between TMP at 18-hour on shorted circuit lifespan.

## DISCUSSION

This retrospective cohort study demonstrates that a higher BFR of 250 ml/min, compared to 200 ml/min, serves as a protective strategy to enhance circuit longevity and might potentially improve clinical outcomes in anticoagulation-free CRRT. A higher BFR was associated with significantly prolonged circuit lifespan. It also increased the percentage of patent circuits reaching preset time milestones. Higher substitution rate, hyperlipidemia, and Hct over 0.3 were identified as risk factors, whereas higher BFR, use of ST150 filter, concomitant with anticoagulation agents for non-CRRT purposes, and prolonged APTT were protective factors of circuit survival. Moreover, patients in the 250 ml/min group showed lower in-hospital mortality. TMP was significantly reduced in the 250 ml/min group than compared with that in the 200 ml/min group, which might play a mediating role in the protection of higher BFR on circuit longevity.

The successful implementation of CRRT necessitates maintaining circuit patency, which largely relies on anticoagulant administration. However, anticoagulation-free CRRT remains clinically indispensable in populations with significant bleeding risks. A network meta-analysis has demonstrated substantially longer circuit lifespans with standard anticoagulants, including 37 hours with RCA, 41.3 hours with low-molecular-weight heparin, and 37.7 hours with Nafamostat Mesylate [15]. Conversely, anticoagulation-free CRRT inherently exhibits shorter circuit durations, commonly ranging from 12 to 32 hours according to previous studies [6, 10], which aligns with our finding with a median circuit lifespan of around 20 hours. While the literature recognizes that CRRT circuit longevity is influenced by multiple factors, including patient characteristics, underlying pathophysiology, vascular access selection, circuit parameters, and clinician expertise, modifiable non-pharmacological strategies remain understudied in anticoagulation-free settings [16, 17]. As a controllable parameter, BFR has been hypothesized to influence circuit duration by minimizing circuit clotting [11, 13, 14]. Yet, notable international variation in BFR settings and inconsistent empirical outcomes underscore the complexity of this relationship. Previously conducted randomized controlled trials (RCTs) offer conflicting evidence. One trial in 2000 reported no difference in time to clot between the standard BFR (125 ml/min) group and augmented BFR (200–250 ml/min) group [18]. Similarly, a subsequent RCT involving 462 circuits distributed between groups of 150 ml/min and 250 ml/min reported comparable clotting risks and median circuit durations (9.1 versus 10 hours; *P* = .37) between groups [19]. Conversely, Dunn *et al*. analyzed data from 1332 treatment sessions involving 355 patients, demonstrating significantly reduced filter lifespan with BFR below 200 ml/min, and identifying an optimal BFR range of 250–300 ml/min to extend circuit longevity [13]. Given these discrepancies, the hypothesis that elevated BFR prolongs circuit duration warrants further empirical validation, particularly within anticoagulation-free CRRT settings. More recently, Zhang *et al*. investigated circuit lifespan in anticoagulation-free CRRT at a fixed BFR of 200 ml/min, reporting a median filter lifespan of 21.5 hours; however, when focusing exclusively on clotted circuits, their observed median duration (16 hours) closely parallels our finding of 15.5 hours [20]. Further rigorous research specifically targeting anticoagulation-free CRRT and different BFR settings remains critical to substantiate optimal clinical practices.

Our study represents one of the first investigations examining CRRT circuit lifespan at different BFR levels in an anticoagulation-free context. In this study, the absolute values of AP and VP were observed to be higher at a BFR of 250 ml/min, whereas TMP was comparatively lower. Notably, TMP demonstrated a negative correlation with clotted circuit lifespan—a relationship which strengthened progressively over the duration of CRRT. Elevated TMP has been widely considered an early warning signal of imminent circuit clotting, given its role in promoting concentration polarization on membrane surfaces, thus impeding membrane permeability. Supporting this theory, several previous studies have established an association between elevated TMP and shortened circuit lifespan. Düngen *et al*. reported reduced TMP (106 mmHg vs. 194 mmHg) extended circuit survival time (1276 min vs. 851 min) when utilizing filters with longer hollow fibers [21]. Additionally, a meta-analysis identified an inverse relationship between BFR and TMP, highlighting their concurrent impacts on circuit longevity [22]. Similarly, a retrospective cohort study involving 26 pediatric patients reported a 1.5% increase in clotting risk for each 1 mmHg elevation in TMP [23]. Prompted by these findings, we conducted a mediation analysis to elucidate potential mechanistic connections and identified that the beneficial effect of higher BFR on prolonging circuit life was at least partially mediated via reductions in TMP. However, whether TMP dynamically responds to changes in BFR in a causal sequence remains unclear. Clarifying this relationship may help validate TMP as a mechanistic intermediary in prolonging circuit life.

Beyond anticoagulation strategies and BFR settings, multiple clinical and technical variables substantially influence CRRT circuit longevity. Prolonged APTT has been consistently associated with diminished circuit clotting risk [19], with each 1-second increase conferring 17% higher filter survival odds [16]. It is noteworthy that Hct ≥0.3 emerged as an independent predictor of circuit failure in our study, corroborating findings from existing literature reporting a significant association between Hct and circuit life [24]. Mechanistically, we speculated that elevated Hct increases blood viscosity, augmenting resistance across the filter and slowing cellular transit, thus promoting coagulation. This pathophysiological explanation is reinforced by pre-dilution’s protective effect through rheological Hct reduction [25]. Several studies have examined the impact of dilution mode on circuit longevity. In a randomized crossover trial, post-dilution continuous venous-venous hemofiltration (CVVH) was associated with a shorter filter life, compared to pre-dilution [26]. Another study conducted in our center suggested that a combined pre- to post-dilution strategy may further prolong circuit lifespan while maintaining solute control, in comparison with the pre-dilution and post-dilution modes during CVVHDF [27]. Hyperlipidemia independently shortened circuit survival in anticoagulation-free CRRT, in agreement with reports of triglyceride-induced circuit occlusion in COVID-19 patients [28]. Some cases observed lipid-containing substances exuded from the fibers, leading to fibrin deposition and subsequent occlusion of the hollow fibers [29, 30]. In addition, the FF has also been identified as another critical determinant of filter patency. An elevated FF reflects a higher post-filter Hct, thereby promoting hemoconcentration and filter clotting [2]. Previous studies demonstrated that an FF exceeding 0.25 is associated with reduced filter performance and a higher risk of premature circuit failure [2, 31]. In the present study, the FF was 0.15 in the low-BFR group and 0.09 in the high-BFR group (*P *< .001), indicating a relatively lower hemoconcentration tendency in patients receiving higher BFR. Interestingly, even after adjusting FF as a covariate in the multivariate Cox regression model, the association between higher BFR and prolonged circuit lifespan remained statistically significant. This finding suggests that mechanisms beyond hemoconcentration are involved in the beneficial effect of higher BFR on filter patency. Mechanistically, higher BFR likely improves intrafilter hemodynamics by reducing blood stasis and shear stress heterogeneity, which may attenuate platelet activation and clot formation [2, 32], and a higher flow rate also stabilizes blood viscosity and delays TMP elevation, collectively contributing to longer circuit survival [23, 33]. Besides, higher substitute speed might lead to an elevated FF, thereby potentially reducing circuit lifespan. Collectively, these findings emphasize the complexity of circuit lifespan determinants in CRRT, especially when pharmacologic anticoagulation is contraindicated. Our results advocate for a multifaceted approach incorporating vigilant monitoring and proactive management of hematological parameters (e.g. Hct and coagulation profiles), lipid profiles, and optimized operational parameters for prolonging CRRT efficacy in high-risk populations. Further studies are needed to confirm causality and elucidate underlying mechanisms.

Effective blood purification delivery has been associated with better clinical results. A retrospective study pointed out that in comparison with patients receiving anticoagulant-free CRRT, those with RCA exhibited significantly lower mortality [34]. Another study elucidated the impact of BFR on all-cause mortality among patients undergoing hemodialysis. It enrolled 1129 prevalent hemodialysis patients and categorized them based on a median BFR threshold of 250 ml/min. K–M analysis revealed significantly higher mortality rates in the group with BFR <250 ml/min compared to those with BFR ≥250 ml/min. Importantly, this association persisted after multivariate adjustment [35]. Our study observed that a BFR of 250 ml/min, compared to 200 ml/min, was associated with improved patient survival even in a fully adjusted model. Higher BFR increases the concentration gradient between blood and dialysate while simultaneously shortening blood residence time within the filter, facilitating improved diffusive clearance of solutes, particularly small molecular weight substances. Efficient solute removal and improved hemodynamic stability at higher BFRs may better maintain inner environment homeostasis. In addition, uninterrupted therapy with long circuit lifespan may lead to more consistent metabolic and volume control, which is crucial in critically ill patients. Nevertheless, given our relatively modest sample size of 128 patients, larger prospective studies are warranted to confirm these observed protective effects.

We acknowledge several limitations in this study. First, its retrospective, single-center design may affect the generalizability of our findings. The limited sample size prevented the use of propensity score matching to mitigate potential selection bias and constrained the robustness of conclusions. Second, the exclusion of CRRT sessions that did not maintain the initially prescribed BFR throughout treatment may introduce bias toward patients with better vascular access and hemodynamic stability, potentially overestimating the benefits of higher BFR. As BFR is often adjusted dynamically in clinical practice, our findings may not fully reflect real-world CRRT scenarios. Moreover, the impact of BFR fluctuations on AP, VP, and TMP was not continuously monitored, highlighting the need for future studies incorporating real-time BFR tracking. Third, our analysis did not include other commonly used BFRs, such as 150 ml/min. Since BFR assignment was based on clinician discretion rather than randomization, future RCTs are warranted to minimize allocation bias and validate these findings [36]. Fourth, the observed trend toward better in-hospital mortality should be interpreted with caution due to the lack of adjustment for baseline disease severity scores, such as Sequential Organ Failure Assessment and Acute Physiology and Chronic Health Evaluation II (APACHE-II). Finally, our exploration of how BFR affects circuit longevity through TMP modulation remains hypothesis-generating rather than conclusive. Focused studies are needed to elucidate the underlying hemodynamic and rheological mechanisms.

## CONCLUSION

In anticoagulation-free CRRT, a BFR of 250 ml/min confers a protective effect compared to 200 ml/min, demonstrating a reduction in circuit clotting, prolonged circuit lifespan, and a potential improvement in in-hospital survival. TMP appears to be a key mediator of the extended circuit longevity associated with higher BFR. Future multicenter RCTs are warranted to validate these findings and to define the optimal BFR for diverse patient populations and clinical scenarios.

## Ethics approval

This study was conducted in compliance with the Declaration of Helsinki and received approval from the Biomedical Research Ethics Committee of West China Hospital, Sichuan University (approval no. 2024-918).

## Participant consent

Given the retrospective nature of the study, the requirement for informed written consent was waived.

## Supplementary Material

sfaf360_Supplemental_File

## Data Availability

The data that support the findings of this study are available from the corresponding author upon reasonable request.
