# Supplementary material for: Higher blood flow rates in anticoagulation-free CRRT improve circuit survival and clinical outcome
Source: Clin Kidney J. 2025 Nov 21;19(1):sfaf360. doi: 10.1093/ckj/sfaf360 (PMC12757746; doi:10.1093/ckj/sfaf360)
Supplement: sfaf360_Supplemental_File [file sfaf360_supplemental_file.docx]

Supplementary Material

**SUPPLEMENTARY APPENDIX**

**Table S1**. Logistic and linear regression analyses of the association between the BFR and clinical outcomes adjusted by baseline disease severity.

**Table S2**. Comparisons of AP, VP, and TMP at different timepoints between the two groups.

**Table S3.** Logistic regression analyses of the association between circuit pressures and circuit clotting events

**Table S1**. Logistic and linear regression analyses of the association between the higher BFR and clinical outcomes adjusted by baseline disease severity.

| **Clinical outcomes** | **Model 1** | |  | **Model 2** | |
| --- | --- | --- | --- | --- | --- |
|  | **OR****/β (95%CI)** | **P** |  | **OR/β (95%CI)** | **P** |
| In-hospital death | 0.32 (0.18 ~ 0.60) | <.001 |  | 0.32 (0.17 ~ 0.60) | <.001 |
| Dialysis-dependency | 3.81 (0.96 ~ 15.16) | 0.057 |  | 3.43 (0.79 ~ 14.87) | 0.100 |
| Length of hospitalization | 10.19 (2.59 ~ 17.80) | 0.011 |  | 6.36 (-1.51 ~ 14.24) | 0.119 |

Model 1: unadjusted model

Model 2: adjusted by baseline lactate, vasopressor requirement, and mechanical ventilation

Abbreviations: BFR, blood flow rate; OR, odds ratio

**Table S2**. **Comparisons of AP, VP, and TMP at different timepoints between the two groups.**

| **Variables** | **Total (n = 241)** | **200 mL/min (n = 132)** | **250 mL/min (n = 109)** | **Statistic** | **P** |
| --- | --- | --- | --- | --- | --- |
| AP at peak, mmHg | -117.00 (-152.75, -90.00) | -98.00 (-129.00, -77.00) | -136.00 (-175.00, -112.00) | Z=-7.35 | <.001 |
| AP at 2-hour, mmHg | -80.00 (-110.75, -65.00) | -69.00 (-86.00, -61.00) | -99.00 (-123.00, -76.00) | Z=-5.17 | <.001 |
| AP at 6-hour, mmHg | -91.00 (-119.00, -72.00) | -78.00 (-96.00, -65.00) | -112.00 (-128.00, -91.00) | Z=-6.75 | <.001 |
| AP at 12-hour, mmHg | -98.00 (-121.00, -76.75) | -81.00 (-104.50, -70.50) | -116.00 (-133.00, -93.00) | Z=-5.69 | <.001 |
| AP at 18-hour, mmHg | -98.50 (-124.75, -74.50) | -83.50 (-104.75, -67.00) | -115.00 (-138.50, -90.50) | Z=-4.59 | <.001 |
| VP at peak, mmHg | 102.50 (75.75, 138.25) | 86.50 (67.00, 120.25) | 116.00 (91.00, 156.75) | Z=-5.26 | <.001 |
| VP at 2-hour, mmHg | 72.00 (53.00, 93.00) | 62.50 (50.75, 80.25) | 80.00 (60.00, 109.00) | Z=-4.02 | <.001 |
| VP at 6-hour, mmHg | 75.50 (54.25, 98.75) | 65.00 (50.00, 86.00) | 86.00 (68.00, 114.00) | Z=-4.06 | <.001 |
| VP at 12-hour, mmHg | 78.00 (58.00, 99.00) | 62.00 (55.00, 85.00) | 86.00 (67.00, 106.00) | Z=-3.93 | <.001 |
| VP at 18-hour, mmHg | 76.00 (58.00, 99.00) | 62.00 (55.00, 85.00) | 84.50 (70.25, 103.75) | Z=-3.23 | 0.001 |
| TMP at peak, mmHg | 120.00 (75.00, 199.50) | 108.00 (75.00, 198.00) | 126.00 (71.50, 203.50) | Z=-0.22 | 0.828 |
| TMP at 2-hour, mmHg | 48.00 (37.00, 58.00) | 56.00 (45.75, 63.00) | 39.00 (28.00, 48.00) | Z=-7.69 | <.001 |
| TMP at 6-hour, mmHg | 53.00 (43.00, 66.00) | 61.00 (50.00, 72.00) | 44.50 (36.75, 54.00) | Z=-7.11 | <.001 |
| TMP at 12-hour, mmHg | 56.00 (44.00, 68.00) | 64.00 (51.50, 76.00) | 47.00 (39.25, 59.00) | Z=-5.63 | <.001 |
| TMP at 18-hour, mmHg | 59.00 (43.50, 75.00) | 67.00 (59.00, 83.00) | 51.00 (38.00, 67.00) | Z=-4.49 | <.001 |

Abbreviations: AP, arterial pressure; VP, venous pressure; TMP, transmembrane pressure.

**Table S3**. **Logistic regression analyses of the association between circuit pressures and circuit clotting events.**

| **Variables** | **β** | **S.E** | **Z** | **P** | **OR (95%CI)** |
| --- | --- | --- | --- | --- | --- |
| AP at peak | -0.01 | 0.00 | -3.16 | 0.002 | 0.99 (0.99 ~ 0.99) |
| AP at 2-hour | -0.01 | 0.00 | -2.62 | 0.009 | 0.99 (0.98 ~ 0.99) |
| AP at 6-hour | -0.01 | 0.00 | -1.79 | 0.074 | 0.99 (0.98 ~ 1.00) |
| AP at 12-hour | -0.00 | 0.00 | -1.00 | 0.316 | 1.00 (0.99 ~ 1.00) |
| AP at 18-hour | -0.01 | 0.01 | -1.51 | 0.130 | 0.99 (0.98 ~ 1.00) |
| VP at peak | 0.01 | 0.00 | 3.80 | <.001 | 1.01 (1.01 ~ 1.02) |
| VP at 2-hour | 0.00 | 0.00 | 0.53 | 0.599 | 1.00 (0.99 ~ 1.01) |
| VP at 6-hour | 0.00 | 0.00 | 1.47 | 0.141 | 1.00 (1.00 ~ 1.01) |
| VP at 12-hour | 0.01 | 0.00 | 2.36 | 0.018 | 1.01 (1.01 ~ 1.02) |
| VP at 18-hour | 0.00 | 0.00 | 0.41 | 0.685 | 1.00 (0.99 ~ 1.01) |
| TMP at peak | 0.04 | 0.01 | 8.51 | <.001 | 1.05 (1.03 ~ 1.06) |
| TMP at 2-hour | 0.01 | 0.01 | 0.92 | 0.359 | 1.01 (0.99 ~ 1.02) |
| TMP at 6-hour | 0.01 | 0.01 | 1.70 | 0.090 | 1.01 (1.00 ~ 1.02) |
| TMP at 12-hour | 0.02 | 0.01 | 3.36 | <.001 | 1.02 (1.01 ~ 1.04) |
| TMP at 18-hour | 0.03 | 0.01 | 3.55 | <.001 | 1.03 (1.01 ~ 1.05) |

Abbreviations: AP, arterial pressure; VP, venous pressure; TMP, transmembrane pressure.
